# Early pregnancy probiotic supplementation with *Lactobacillus rhamnosus* HN001 may reduce the prevalence of gestational diabetes mellitus: a randomised controlled trial

**DOI:** 10.1017/S0007114517000289

**Published:** 2017-04-03

**Authors:** Kristin L. Wickens, Christine A. Barthow, Rinki Murphy, Peter R. Abels, Robyn M. Maude, Peter R. Stone, Edwin A. Mitchell, Thorsten V. Stanley, Gordon L. Purdie, Janice M. Kang, Fiona E. Hood, Judy L. Rowden, Phillipa K. Barnes, Penny F. Fitzharris, Julian Crane

**Affiliations:** 1 University of Otago, Wellington 6021, New Zealand; 2 University of Auckland, Auckland 1142, New Zealand; 3 Capital and Coast DHB, Wellington 6021, New Zealand; 4 Victoria University, Wellington 6021, New Zealand; 5 Auckland Hospital, Auckland 1142, New Zealand

**Keywords:** Randomised controlled trials, Probiotics, *Lactobacillus rhamnosus* HN001, Gestational diabetes mellitus

## Abstract

The study aims to assess whether supplementation with the probiotic *Lactobacillus rhamnosus* HN001 (HN001) can reduce the prevalence of gestational diabetes mellitus (GDM). A double-blind, randomised, placebo-controlled parallel trial was conducted in New Zealand (NZ) (Wellington and Auckland). Pregnant women with a personal or partner history of atopic disease were randomised at 14–16 weeks’ gestation to receive HN001 (6×10^9^ colony-forming units) (*n* 212) or placebo (*n* 211) daily. GDM at 24–30 weeks was assessed using the definition of the International Association of Diabetes and Pregnancy Study Groups (IADPSG) (fasting plasma glucose ≥5·1 mmol/l, or 1 h post 75 g glucose level at ≥10 mmol/l or at 2 h ≥8·5 mmol/l) and NZ definition (fasting plasma glucose ≥5·5 mmol/l or 2 h post 75 g glucose at ≥9 mmol/l). All analyses were intention-to-treat. A total of 184 (87 %) women took HN001 and 189 (90 %) women took placebo. There was a trend towards lower relative rates (RR) of GDM (IADPSG definition) in the HN001 group, 0·59 (95 % CI 0·32, 1·08) (*P*=0·08). HN001 was associated with lower rates of GDM in women aged ≥35 years (RR 0·31; 95 % CI 0·12, 0·81, *P*=0·009) and women with a history of GDM (RR 0·00; 95 % CI 0·00, 0·66, *P*=0·004). These rates did not differ significantly from those of women without these characteristics. Using the NZ definition, GDM prevalence was significantly lower in the HN001 group, 2·1 % (95 % CI 0·6, 5·2), *v*. 6·5 % (95 % CI 3·5, 10·9) in the placebo group (*P*=0·03). HN001 supplementation from 14 to 16 weeks’ gestation may reduce GDM prevalence, particularly among older women and those with previous GDM.

Lifestyle factors such as changes in patterns of food consumption with economic development have led to the well-recognised and increasing problems of obesity and associated diseases, including gestational diabetes mellitus (GDM), both in New Zealand (NZ)^(^ ^1^ ^)^ and other developed countries^(^ ^2^ ^)^. Pre-pregnancy overweight and obesity have been shown to account for 46 % of GDM^(^ ^3^ ^)^, with excess weight gain during pregnancy, previous GDM or a family history of diabetes, polycystic ovary syndrome (PCOS), older age and higher parity also identified as risk factors^(^ ^4^ ^)^. GDM itself increases the risk for preeclampsia, miscarriage, preterm birth, macrosomia, induction of labour and caesarean section^(^ ^2^ ^,^ ^3^ ^)^. GDM also increases the risk for later maternal and child obesity and subsequent type 2 diabetes mellitus^(^ ^5^ ^)^.

GDM definitions are variable, and establishing an international consensus on diagnostic criteria that predict adverse pregnancy outcomes has been challenging. In 2008, the International Association of Diabetes and Pregnancy Study Group (IADPSG)^(^ ^6^ ^)^ used data from the Hyperglycemia and Adverse Pregnancy Outcome (HAPO) study^(^ ^7^ ^)^ to develop recommendations for oral glucose tolerance test (GTT) threshold glucose concentrations for the diagnosis of GDM (fasting plasma glucose ≥5·1 mmol/l or 1 h post 75 g glucose level ≥10 mmol/l or at 2 h ≥8·5 mmol/l). This was based on the findings of the HAPO study^(^ ^7^ ^)^ of a curvilinear dose–response relationship between fasting, 1 and 2 h glucose concentrations and adverse pregnancy outcomes, including macrosomia and caesarean section delivery. However, the NZ guideline definitions for GDM diagnosis specify a higher baseline and 2 h glucose test threshold (fasting plasma glucose ≥5·5 mmol/l or 2 h post 75 g glucose level ≥9 mmol/l)^(^ ^1^ ^)^.

The WHO has defined probiotics as live micro-organisms, which when administered in adequate amounts confer a health benefit to the host^(^ ^8^ ^)^. There is emerging evidence for a modulating effect of probiotics on gut microbiota and inflammatory responses^(^ ^9^ ^,^ ^10^ ^)^, with a recent meta-analysis suggesting that probiotics can favourably influence glucose metabolism^(^ ^11^ ^)^. A Finnish trial^(^ ^12^ ^)^ showed that a probiotic supplement containing *Lactobacillus rhamnosus* GG and *Bifidobacterium lactis* Bb12, taken from the first trimester of pregnancy, reduced the prevalence of GDM.

The aim of the current study was to investigate whether the probiotic *L. rhamnosus* HN001 (HN001) taken by pregnant mothers from early pregnancy could reduce the prevalence of GDM by 26–28 weeks’ gestation.

## Methods

### Study design

The study was a two-centre, double-blind, randomised, placebo-controlled parallel trial investigating the effects of the probiotic HN001 on the prevalence of GDM. This secondary outcome was decided *a priori.* The primary outcome was the development of eczema and atopic sensitisation in the child at age 12 months (Australia NZ Clinical Trials Registry: ACTRN12612000196842).

For a detailed description of study methods and outcomes refer to Barthow *et al.* ^(^ ^13^ ^)^.

### Participants

In brief, pregnant women in Auckland and Wellington, NZ, were recruited into the study via health professionals and study information placed in pregnancy packs. Women were considered eligible if they were <16 weeks’ gestation, English-speaking, had intention to breast-feed, and if either they or the unborn child’s biological father had a history of asthma, hayfever or eczema requiring medication. Women were excluded from the study if aged <16 years, were planning to move outside the study centres during the study duration, had a history of immunological disorders or medication, or cardiac valve disease, required *in vitro* fertilisation, had major fetal abnormalities, were using probiotic drinks or supplements, participating in another randomised controlled trial, refused notification of their clinical carers, carried adrenaline for cows’ milk allergy, had a history of a transplant or HIV, had used continuous antibiotic therapy for at least 3 months, miscarried between screening and enrolment, or were otherwise deemed unsuitable. Eligible women were enrolled into the study at 14–16 weeks’ gestation, where gestation was determined on the basis of the earliest first-trimester scan and, where this was not available, the date of the last menstrual period.

### Study capsules

Participating women were randomised to receive capsules containing either HN001 (6×10^9^ colony-forming units (cfu)) or placebo (maize-derived maltodextrin, identical in appearance and smell to the probiotic) to be taken daily from enrolment throughout pregnancy and until 6 months post birth if still breast-feeding. HN001 powder was manufactured by Fonterra Co-operative Group Ltd (Fonterra) using aseptic fermentation, concentration and freeze-drying, as previously described^(^ ^14^ ^)^. The placebo powder, maize-derived maltodextrin, was manufactured by Grain Processing Corporation. Women were instructed to keep the capsules in a refrigerator and to avoid taking them within 10 min of consuming hot food or fluid.

Fonterra retained samples of capsules at 4°C, which were tested monthly to ensure viability of the contents over time. The viability of the contents of a selection of unused capsules returned from the field was tested 3 monthly. Loss in viability was <0·1 log, and within the limit of uncertainty of the counting method.

Randomisation of capsules was performed by a statistician at Fonterra who had no contact with the study investigators or participants. Randomisation was stratified by the study centre and performed in blocks of twenty according to a computer-generated randomisation schedule and an allocation ratio of 1:1. Research staff screened and enrolled participants, providing eligible participants with the next available sequentially numbered capsule container. All researchers, laboratory staff and participants were blinded to study allocation.

### Baseline

Information collected included age, ethnicity, parity, previous PCOS, BMI (weight (kg)/height (m^2^)), waist circumference, antibiotic use during pregnancy but before enrolment and type 2 diabetes mellitus in the participant or a first-degree relative. Among women with previous pregnancies >20 weeks, we also collected a history of previous GDM and birth weight of previous babies.

### Outcomes

The GDM outcome was defined *a priori* primarily as the diagnosis of GDM according to the IADPSG recommendations^(^ ^6^ ^)^: a fasting plasma glucose ≥5·1 mmol/l, or 1 h post 75 g glucose load ≥10 mmol/l or at 2 h ≥8·5 mmol/l. A secondary analysis was conducted using NZ thresholds ≥5·5 mmol/l fasting or ≥9 mmol/l at 2 h to define GDM^(^ ^1^ ^)^.

The assessment for GDM was conducted at 24–30 weeks’ gestation following a 12 h overnight fast, using a GTT undertaken at a community laboratory. Only women without pre-pregnancy diabetes were invited to undertake a study GTT. Women who received a GTT-based diagnosis of GDM before the study GTT were included in the study outcome only if there was evidence of earlier negative tests confirming that their diabetes was gestational. When repeat GTTs were performed later in pregnancy (for clinical purposes), the test completed at 24–30 weeks’ gestation determined their study GDM status.

Women with GDM were asked to have a postpartum HbA1_c_ measured at least 3 months after birth. A postpartum HbA1_c_ level ≥6·5 % (48 mmol/mol) or a postpartum fasting glucose ≥7 mmol/l and/or 2 h glucose ≥11·1 mmol/l was used to indicate the presence of co-existing type 2 diabetes. If women met these criteria they were excluded from the GDM analysis.

We collected other outcomes at 4–7 d post birth, including maternal weight (kg) and waist circumference (cm), gestation (in weeks) and prematurity (<37 weeks). Infant Apgar score at 5 min and birth weight were collected from medical records. Infant length (cm), ponderal index (PI) (birth weight (kg)/length (m^3^)), head circumference (cm), type of delivery (vaginal or caesarean) and admission to the neonatal intensive care unit (NICU) were assessed by the researcher at 4–7 d post birth.

### Adherence

More than 3 months’ supply of capsules (*n* 105) were placed in each bottle. Bottles were replaced at 26–28 weeks’ gestation and birth, at which time two bottles were given to the mother to cover the period up to 6 months post birth. Returned capsules were counted by staff not involved in the study assessments, and adherence rates (number taken/time period) were calculated.

### Power

Assuming a 15 % prevalence of GDM, a 63 % reduction due to the probiotic, as found in a Finnish study^(^ ^12^ ^)^, and a sample size of 195 in each group, the study would have 87 % power at the 5 % level of significance.

### Statistical analysis

Analysis was performed using SAS 9.3 and 9.4 (SAS Institute). All analyses were intention-to-treat. Differences between the treatment groups in the prevalence of GDM and dichotomous birth outcomes were estimated using relative rates (RR) and 95 % CI. Although not pre-specified, we conducted an analysis of GDM stratified by factors that were significantly associated with GDM (maternal age, BMI, a history of GDM) and by antibiotic use during the study before the GTT using a generalised linear model with a log-link and binomial distribution. For continuous variables, differences between treatment groups are reported as differences in means (95 % CI) compared using *t* tests, or as ratios of geometric means (95 %CI) compared using ANCOVA on logged values, adjusted for logged baseline measures; other differences were compared with Wilcoxon’s rank-sum tests. The Apgar score was compared between groups using ordinal logistic regression. Tests were two-sided and *P*<0·05 was considered statistically significant.

Missing GTT measurements were estimated with 1000 multiple imputations using treatment, fasting, 1 and 2 h measurements, ethnicity, age, BMI at enrolment, a family history of diabetes in first-degree relatives, previous PCOS, and the combination of previous GDM and number of previous pregnancies of >20 weeks’ gestation (grouped as no previous pregnancies, one previous pregnancy with GDM or one previous pregnancy without GDM, two or more previous pregnancies with GDM or two or more previous pregnancies without GDM). One participant who did not undergo the GTT because she had been diagnosed with GDM and prescribed insulin from early pregnancy was assumed to have GDM in all imputations.

This study was conducted according to the guidelines laid down in the Declaration of Helsinki, and all procedures involving human subjects were approved by the Multi-Region Health and Disability Ethics Committee. Written informed consent was obtained from all subjects. The trial was registered at the Australia NZ Clinical Trials Registry: ACTRN12612000196842, https://www.anzctr.org.au/Trial/Registration/TrialReview.aspx?id=362049&isReview=true.

## Results

**Fig. 1:**
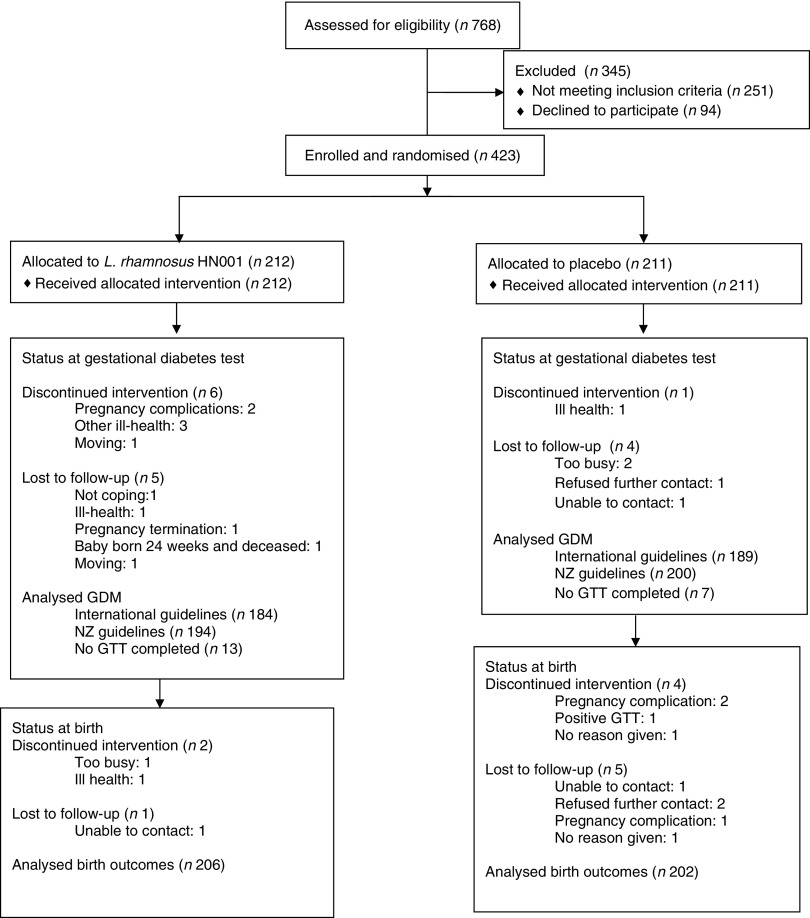
Status of study participants through the trial. GDM, gestational diabetes mellitus; NZ, New Zealand; GTT, glucose tolerance test.

Participants (*n* 423) were randomised to the HN001 (*n* 212) or placebo group (*n* 211) between December 2012 and November 2014 at an average rate of 4·2 a week. Gestational diabetes assessments were completed by February 2015 and the final infant was born in May 2015. Loss-to-follow-up rates were similar between study groups, but more participants in the HN001 group had discontinued the intervention before the GTT (Fig. 1). In contrast, most participants lost to follow-up between the GTT and birth visits were in the placebo group, with small numbers discontinuing the intervention in both study groups. There was one maternal death in the placebo group due to a subarachnoid haemorrhage.

Median adherence rates were 94·9 % (interquartile range (IQR) 85·7–98·8 %) (*n* 179) in the HN001 group and 94·0 % (IQR 85·9–98·8 %) (*n* 183) in the placebo group (Wilcoxon’s rank-sum test, *P*=0·59).

Among randomised participants, the 24–30-week GTT results included all three time points (fasting, 1 h and 2 h values, as required by IADPSG guideline definitions) in 184 (87 %) participants in the HN001 group and 189 (90 %) in the placebo group, at mean 27·7 (sd 4·6) and 28·0 (sd 8·6) weeks’ gestation, respectively. An additional ten HN001 participants and eleven placebo participants had only the fasting and 2-h time-point GTT results available, which were sufficient for a diagnosis of GDM by standard NZ guidelines. A total of 194 (92 %) in the HN001 group and 200 (95 %) in the placebo group participated in either GTT assessment, all of whom were able to contribute data to the analysis on the basis of NZ guidelines.

There were no substantial differences between the study groups in any maternal characteristic at enrolment, including age, ethnicity, parity, weight, waist, BMI, antibiotic use or corticosteroid use during pregnancy before enrolment, family history of diabetes, household income, maternal smoking or maternal treated allergic disease (asthma, eczema or hayfever), and among those with previous births, weight of largest infant and having a history of GDM (Table 1).

**Table 1:** Characteristics of the study population at enrolment (Numbers and percentages; medians and interquartile ranges (IQR))

	HN001 (*n* 212)		Placebo (*n* 211)	
	%	*n*	%	*n*
Previous pregnancy	67·5	143/212	73·8	155/210
Diabetes in first-degree relative	17·9	38/212	18·5	39/211
Previous polycystic ovary syndrome	8·1	17/209	10·6	22/208
Age (years)	*n* 212		*n* 210	
Median	34		34	
IQR	30–36		31–37	
Weight (kg)	*n* 211		*n* 210	
Median	69		71	
IQR	63–80		63–82	
Waist circumference (cm)	*n* 211		*n* 210	
Median	87		87	
IQR	80–94		81–99	
BMI (kg/m^2^)	*n* 211		*n* 210	
Median	25		26	
IQR	23–29		23–30	
Ethnicity		212		211
Maori	10·9	23	16·6	35
Pacific	3·8	8	1·9	4
Asian	7·7	15	7·6	16
European	78·3	166	73·5	155
Other	0·0	0	0·5	1
Systemic antibiotic use during pregnancy before enrolment	12·4	25/201	13·9	28/202
Smoking	5·2	11	3·8	8
Systemic corticosteroid use	1·4	3	1·4	3
Treated asthma, eczema or hayfever	87·3	185	82·9	175
Household income (NZ$)	207		204	
0–49 000	8·2	17	7·8	16
50–99 000	30·4	63	32·4	66
100–149 000	35·8	74	34·8	71
150 000+	25·6	53	25·0	51
Previous gestational diabetes	5·0	6/121	7·1	9/127
Weight of previous largest baby (g)	*n* 119		*n* 124	
Median	3520		3547	
IQR	3260–3900		3232–3856	
Macrosomia (previous largest baby≥4000 g)	20·2	24/119	14·5	18/124

HN001, *Lactobacillus rhamnosus* HN001.

The prevalence of GDM (defined using the IADPSG criteria) in the HN001 group was lower than that in the placebo group, but this difference was not statistically significant (Table 2). However, using the more specific NZ definition, the prevalence of GDM was significantly lower in the HN001 group. There were three participants in the HN001 group and five in the placebo group using oral or injected corticosteroids between enrolment and the GTT (*P*=0·48). Among this small number of participants there was no increased risk for gestational diabetes (data not shown). Adjustment for systemic corticosteroid use during this time period made only minimal differences to the treatment effects on gestational diabetes for both the IADPSG (RR 0·59; 95 % CI 0·32, 1·08) and the NZ (RR 0·31; 95 % CI 0·10, 0·95) guidelines. These analyses were repeated with imputed results for missing values but there was little change in the RR estimates. Using the IADPSG guidelines the imputed RR=0·64 (95 % CI 0·36, 1·11), and using the NZ guidelines imputed RR=0·33 (95 % CI 0·11, 0·99).

**Table 2:** Treatment effects on the prevalence of gestational diabetes mellitus defined according to International Association of Diabetes and Pregnancy Study Groups (IADPSG)* and New Zealand (NZ)† definitions, and mean blood glucose levels (Prevalence percentages and 95 % confidence intervals; relative rates (RR) and 95 % confidence intervals; mean values and 95 % confidence intervals)

	HN001		Placebo					
	Prevalence (%)	95 % CI (%)	Prevalence (%)	95 % CI (%)	RR	95 % CI	*P*	*P* (multiple imputation)
IADPSG* (*n* 373)								
	8·2 (15/184)	4·6, 13·1	13·8 (26/189)	9·2, 19·5	0·59	0·32, 1·08	0·08	0·12
NZ† (*n* 394)								
	2·1 (4/194)	0·6, 5·2	6·5 (13/200)	3·5, 10·9	0·32	0·11, 0·96	0·03	0·07
	Mean	95 % CI	Mean	95 % CI	Difference in mean	95 % CI		
Fasting (mmol/l)	*n* 195		*n* 202					
	4·32	4·27, 4·37	4·40	4·34, 4·46	−0·08	−0·15, 0·00	0·048	0·06
1 h (mmol/l)	*n* 185		*n* 189					
	6·71	6·46, 6·96	6·89	6·63, 7·15	−0·18	−0·55, 0·18	0·31	0·42
2 h (mmol/l)	*n* 194		*n* 200					
	5·65	5·47, 5·83	5·78	5·57, 5·99	−0·13	−0·41, 0·15	0·36	0·39

HN001, *Lactobacillus rhamnosus* HN001.

* Fasting ≥5·1 mmol/l, 1 h≥10 mmol/l, 2 h≥8·5 mmol/l.

† Fasting ≥5·5 mmol/l, 2 h≥9 mmol/l.

The mean blood glucose levels at baseline and after 1 and 2 h were slightly lower in the HN001 group compared with the placebo group, but were significant only at baseline (Table 2).

Among forty-four participants diagnosed with GDM according to either the NZ or IADPSG guidelines, forty participants had HbA1_c_ measured between 1 and 15 months post birth, with values between 5·0 % (31 mmol/mol) and 6·4 % (46 mmol/mol). One participant had a post-birth GTT, with values within the normal range. Three participants did not have post-birth HbA1_c_ measured because one had declined, one had withdrawn from the study, and one had died.

**Fig. 2:**
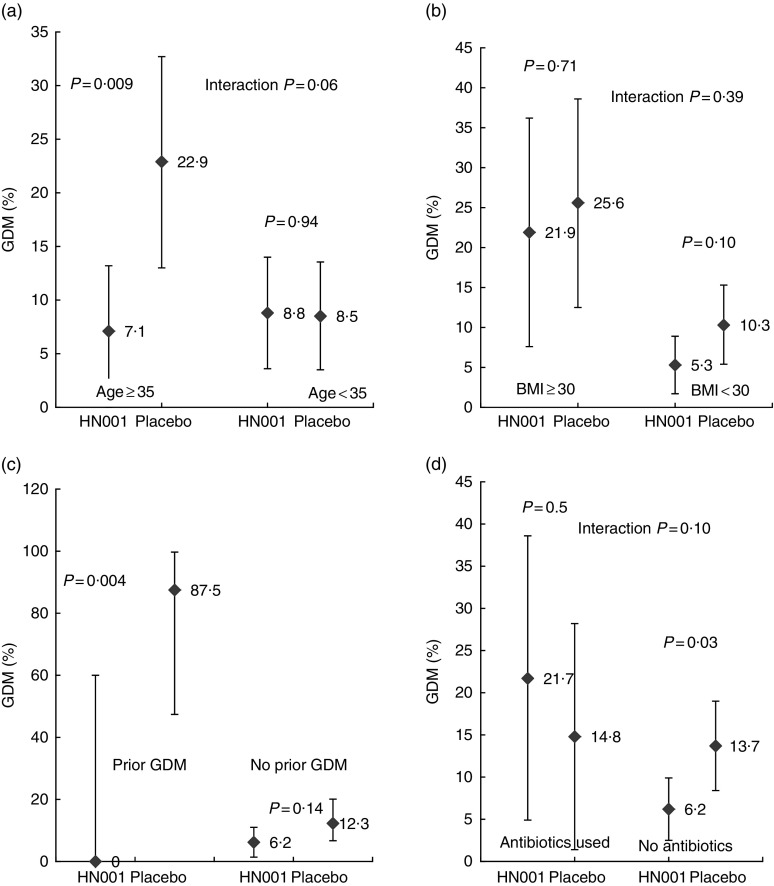
*Lactobacillus rhamnosus* HN001 (HN001) associations with gestational diabetes mellitus (GDM) stratified by (a) age ≥35 *v*. <35 years, (b) BMI≥30 *v*. <30 kg/m^2^, (c) history of GDM and (d) systemic antibiotic use since enrolment.

Table 3 shows that only maternal age, BMI and having a history of GDM were significantly associated with GDM in this study. These factors were then used to stratify the analysis (Fig. 2) using only the IADPSG definition of GDM, as this definition reflects our *a priori* hypothesis. There was a significant treatment-by-age (as a continuous variable) interaction (*P*=0·005) and a non-significant interaction with age dichotomised as ≥35 *v*. <35 years (*P*_interaction_=0·06). In the older group, HN001 was associated with a 3-fold reduction in the prevalence of GDM compared with the prevalence among women in the placebo group (RR 0·31; 95 % CI 0·12, 0·81, *P*=0·009). In women aged <35 years the prevalence in each study group was similar (RR 1·04; 95 % CI 0·45, 2·39). There were also no significant differences in effect dependent on whether BMI was ≥30 kg/m^2^ (RR 0·86; 95 % CI 0·37, 1·96), or <30 kg/m^2^ (RR 0·51; 95 % CI 0·22, 1·17) (*P*_interaction_ 0·39). GDM did not recur in any of the HN001 participants who had a history of GDM. Thus, we could not test for an interaction effect. Among those with a history of GDM, HN001 protected against a recurrence of GDM, RR=0·00 (95 % CI 0·00, 0·66), and for those without previous GDM, RR=0·50 (95 % CI 0·20, 1·27). Three women (20 %) with a history of GDM did not complete the GTT according to the IADSPG guidelines. In all imputations, these were GDM positive, giving an imputed RR=0·38 (95 % CI 0·05, 1·00, *P*=0·043, Barnard’s exact test). As HN001 is susceptible to a range of antibiotics^(^ ^15^ ^)^, we examined the effect of HN001 on GDM by use of antibiotics between study enrolment and the GTT test. The HN001 effect on GDM was significantly protective among participants who had not used antibiotics between study enrolment and the GTT test but there was no significant effect of HN001 for those who had used antibiotics during this period (*P*_interaction_=0·10).

**Table 3:** Association of maternal risk factors with gestational diabetes mellitus, after adjustment for treatment group (Relative rates (RR) and 95 % confidence intervals)

	*n*	RR	95 % CI	*P*
Maternal age at enrolment*	372	1·11	1·03, 1·20	0·004
Maternal BMI at enrolment (kg/m^2^)†	371	1·11	1·08, 1·15	<0·0001
Family history of diabetes	67/373	1·13	0·55, 2·33	0·74
Ethnicity	373			
European	293	1·00		
Maori	46	1·49	0·69, 3·22	0·31
Pacific	7	3·05	0·92, 10·07	0·07
Asian	26	1·62	0·62, 4·22	0·32
Polycystic ovary syndrome	37/367	1·19	0·50, 2·83	0·70
Systemic antibiotic use since enrolment	49/372	1·78	0·91, 3·49	0·09
Previous pregnancy (of any duration)	259/372	1·30	0·66, 2·56	0·45
Among women with any previous pregnancy (259/372)				
Number of miscarriages‡	259	1·12	0·78, 1·62	0·54
Previous pregnancy >20 weeks	216/372	1·24	0·68, 2·26	0·47
Among women with previous pregnancies ≥20 weeks (216/372)				
Previous gestational diabetes	12/216	6·59	3·95, 11·01	<0·0001
Macrosomia§ in previous child	35/213	0·66	0·19, 2·35	0·53

* For each additional year of age.

† For each additional BMI unit; two values >45 truncated at 45 to ensure model fit.

‡ For each additional miscarriage.

§ Birth weight ≥4000 g.

Similarly, among those not using antibiotics, fasting mean blood glucose levels were significantly lower (*P*=0·001) in the HN001 group (4·28; 95 % CI 4·23, 4·33) compared with that in the placebo group (4·42; 95 % CI 4·35, 4·48). Differences in mean glucose levels at 1 and 2 h post glucose load were also lower but were not significant. At 1 h, blood glucose levels were 6·63 (95 % CI 6·37, 6·89) in the HN001 group and 6·88 (95 % CI 6·60, 7·16) in the placebo group (*P*=0·20), and at 2 h the levels were 5·56 (95 % CI 5·38, 5·73) in the HN001 group and 5·77 (95 % CI 5·53, 6·01) in the placebo group (*P*=0·15).

HN001 was not significantly associated with any maternal anthropometric measures (after adjustment for baseline measurements), or infant birth weight, gestation, caesarean delivery or admission to the NICU or, at 4–7 d post birth, infant length, PI or head circumference. Infants whose mothers were in the HN001 group had a significantly higher 5-min Apgar score than did infants in the placebo group (Table 4).

**Table 4:** Treatment effects on birth outcomes (Geometric means and 95 % confidence intervals; median and interquartile range (IQR); mean values and 95 % confidence intervals; relative rates (RR) and 95 % confidence intervals)

	HN001		Placebo				
	Geometric mean	95 % CI	Geometric mean	95 % CI	Geometric mean ratio	95 % CI	*P*
Maternal weight post birth (kg)*	*n* 197		*n* 194				
	76·7	76·1, 77·2	76·8	76·2, 77·4	1·00	0·99, 1·01	0·79
Maternal waist post birth (cm)*	*n* 195		*n* 195				
	97·7	96·8, 98·7	97·3	96·4, 98·2	1·00	0·99, 1·02	0·53
Maternal BMI post birth (kg/m^2^)*	*n* 197		*n* 194				
	28·0	27·8, 28·2	28·1	27·9, 28·3	1·00	0·99, 1·01	0·69
Number of weeks of gestation	*n* 205		*n* 201		Difference in median		
Median	39·7		39·6		0·1	−0·1, 0·4	0·31†
IQR	38·7, 40·7		38·7, 40·4				
	Mean	95 % CI	Mean	95 % CI	Differences in means	95 % CI	
Birth weight of child (kg)	*n* 205		*n* 202				
	3·6	3·5, 3·7	3·5	3·4, 3·6	0·1	−0·1, 0·2	0·36
Birth length of child (cm)	*n* 205		*n* 199				
	51·3	51·0, 51·7	51·2	50·8, 51·5	0·2	−0·3, 0·7	0·48
Ponderal index of child	*n* 204		*n* 199				
	25·9	25·5, 26·2	25·7	25·4, 26·1	0·1	−0·4, 0·7	0·59
Head circumference of child (cm)	*n* 205		*n* 201				
	35·3	35·1, 35·6	35·4	35·2, 35·6	−0·1	−0·4, 0·2	0·67
	%	*n*	%	*n*	RR	95 % CI	
Macrosomia (≥4000 g)	22·4	46/205	15·8	32/202	1·41	0·94, 2·12	0·10
Premature (<37 weeks’ gestation)	7·8	16/205	4·0	8/201	1·96	0·86, 4·48	0·10
Caesarean delivery	27·7	57/206	25·4	51/201	1·09	0·79, 1·51	0·60
Admission to NICU	11·3	23/203	11·0	22/201	1·04	0·60, 1·80	0·90
Apgar score ≥7 at 5 min	98·5	200/203	98·0	198/202	1·51‡	1·01, 2·27	0·04

HN001, *Lactobacillus rhamnosus* HN001; NICU, Neonatal Intensive Care Unit.

* ANCOVA on logged values, adjusted for logged baseline measures, geometric means are fitted for the baseline geometric mean.

† Wilcoxon’s rank-sum test.

‡ OR of having a higher Apgar score; Apgar scores grouped 0–3, 4–6, 7, 8, 9, 10: ordinal logistic regression.

GDM in the mother, defined according to the IADPSG recommendations, was associated with higher maternal weight (*P*=0·0002), waist circumference (*P*<0·0001) and BMI (*P*<0·0001) post birth but was not associated with any infant anthropometric measures, gestation, caesarean delivery, NICU admission or Apgar score at 5 min (data not shown).

## Discussion

As far as we are aware, this is the first study to report a role for probiotics in preventing GDM among women not selected on the basis of risk for GDM. Our data suggest that the probiotic HN001 at a dose of 6×10^9^ cfu/d may lower the rate of GDM from 13·8 to 8·2 %, a 40 % reduction using the IADPSG guidelines^(^ ^6^ ^)^ or a 68 % reduction from 6·5 to 2·1 % using the NZ guidelines. Differences in mean blood glucose levels were small at baseline, and at 1 and 2 h post glucose load. Nevertheless, these differences in absolute values correspond to the difference in GDM prevalence we found using thresholds.

The HN001 protective effect on GDM (defined using the IADPSG criteria) found in this study was weaker than the probiotic effect on GDM found among women at risk for GDM in Finland^(^ ^12^ ^)^, but older women and women with a history of GDM had similar risks to the at-risk Finnish population. This Finnish study intervened from the first trimester of pregnancy with a combination of two probiotics (*L. rhamnosus* GG and *B. lactis* Bb12) and reported a significant reduction in GDM prevalence from 36 to 13 % due to the probiotics. Both active and placebo groups also had a dietary intervention. It is possible that the probiotics interacted with diet to enhance protection against GDM, or that the combination of the two probiotics used in the Finnish study was more effective than HN001 alone. The higher prevalence of GDM in the Finnish trial may be a consequence of limiting the test to an at-risk sub-group and the different diagnostic thresholds applicable in Finland at that time (≥4·8 mmol/l at baseline, ≥10 mmol/l at 1 h, or ≥8·7 mmol/l at 2 h)^(^ ^12^ ^)^. Applying the Finnish at-risk criteria^(^ ^16^ ^)^ to our study population, at least 58 % of the women would have been at risk, and applying the Finnish GDM diagnostic criteria to these women at risk, 27 % of women in the placebo group and 22 % of women in the HN001 group would have been classified as having GDM. In smaller studies, Lindsay *et al*. have shown no significant effect of *Lactobacillus salivarius* UCC118 on fasting glucose levels among obese women when taken between 24 and 28 weeks’ gestation^(^ ^17^ ^)^ or as a 4–6-week treatment for women with either impaired glucose tolerance or GDM^(^ ^18^ ^)^. This may indicate that this particular strain of probiotic is an ineffective intervention for prevention or treatment of GDM, or that the intervention period was too short or too late in pregnancy to observe an effect, or the studies lacked statistical power.

Our study showed that the HN001 effect was stronger using the higher NZ glucose thresholds than the IADPSG thresholds to define GDM, suggesting that the effect is greater in preventing more severe GDM.

Taking one or more systemic antibiotic courses during the same period as the HN001 may negate any effect of HN001 on GDM, with benefits of taking HN001 found only among those who did not require antibiotics. However, because of the small percentage (11 %) taking antibiotics, the study had low power to assess differences in HN001 effect dependent on antibiotic use (*P*=0·10). There was a similar strengthening effect of HN001 on blood glucose levels, particularly at baseline, after excluding those taking antibiotics. These data might indicate the deleterious effect that antibiotics have on gut microbiota composition and function^(^ ^19^ ^)^, possibly compromising the viability of HN001.

The HN001 intervention appeared to have no effect on maternal or infant anthropometric measures post birth. Gestation duration, rate of caesarean delivery and admission to the NICU were also unaffected by HN001, findings that are consistent with other studies^(^ ^12^ ^,^ ^17^ ^)^. One explanation for a lack of effect may be that maternal use of medication or dietary/exercise regimens to reduce blood glucose levels could have obscured any effect of untreated GDM on post-birth measures in the infant or the mother. The effects of antibiotics on the gut microbiota if taken during the third trimester, but before labour, could also have obscured an effect. However, removal of the 13 % using systemic antibiotics during this period had little impact on the associations of HN001 with anthropometric measures. Assuming this discrepancy between an HN001 effect on GDM but not on anthropometric measures at birth is not explained by obstetric interventions to limit excessive weight gain, there may be a mechanism whereby HN001 has an effect on blood sugar levels but not on maternal weight.

There was a significant (*P*=0·04) beneficial effect of HN001 on the 5-min Apgar score, possibly due to chance given the number of comparisons performed.

The gut microbiota is profoundly altered during the three trimesters of pregnancy towards a less diverse state, with the most depleted microbial richness found in women with GDM^(^ ^20^ ^)^. In contrast to obesity-related gut microbiota, the last-trimester gut microbiota has been associated with greater amounts of energy lost in stool compared with the first trimester, indicating that the impact of gut microbiota alterations during pregnancy on host adiposity and host glucose metabolism is not necessarily identical^(^ ^20^ ^)^. We speculate that HN001 supplementation altered the composition and function of the gut microbiota in favour of improved insulin sensitivity and inflammation in the host, which reduced the propensity towards GDM.

The lack of any deleterious effect on birth outcomes supports HN001 as a safe intervention to take from early pregnancy (14–16 weeks’ gestation), which may also be beneficial to the infant, as reflected by the Apgar score. These findings are important given the small amount of data available on effects of early-pregnancy probiotic interventions.

A limitation of our study is that we did not collect maternal anthropometric measures at the time of the GTT. We were also unable to measure pre-pregnancy maternal anthropometric measures, and hence changes in maternal weight and waist circumference during pregnancy were based on measurements taken at enrolment (14–16 weeks’ gestation), when differences may already reflect a change from pre-pregnancy weight. However, women are recommended to gain less than 2 kg in their first trimester^(^ ^21^ ^)^, and many women gain considerably less^(^ ^22^ ^)^. Given the smaller reduction in GDM we found compared with Luoto *et al*.^(^ ^12^ ^)^, we may also have overestimated the power of the study to find a difference by basing it on their findings.

The strengths of this study are the larger sample size and good follow-up rates compared with previous studies, and good generalisability because our population was not selected for high risk for GDM. As underlying low-grade inflammation may be a predisposing factor in obesity, diabetes and allergic disease^(^ ^23^ ^)^, our study population, selected to be at risk for allergic disease, may also be at greater risk for diabetes. Further, systemic corticosteroid use, most likely for asthma management in our study population, may have increased the rate of insulin resistance and gestational diabetes^(^ ^24^ ^)^. However, it is unlikely that these factors explain our study findings, given that the 6·5 % prevalence of GDM in the placebo group is representative of the NZ prevalence^(1)^ and corticosteroid use was not associated with increased gestational diabetes for either GDM definition. The generalisability of the study may also be compromised because women self-selected for study may be better educated and have higher incomes than women not included in the study. However, these factors are unlikely to be associated with an HN001 effect.

We were also able to exclude the possibility that women with undiagnosed type 2 diabetes mellitus were misclassified as GDM by measuring their postpartum HbA1_c_ or GTT. Most of these women (40/44) underwent postpartum evaluation of their glycaemic status but none met a level indicative of type 2 diabetes mellitus^(^ ^25^ ^)^, suggesting that the cases of GDM are likely to be gestational only.

Promoting good health in pregnancy through weight control programmes or diet has been largely ineffective, partly due to poor adherence with the interventions. If our results for probiotics are confirmed in other larger trials, the promise of a simple, cheap and safe intervention is an attractive option to reduce the prevalence of GDM, which is increasing not only in affluent countries but also in less-affluent countries as they become more Westernised.
